# Drought-induced assembly of rhizosphere mycobiomes shows beneficial effects on plant growth

**DOI:** 10.1128/msystems.00354-24

**Published:** 2024-06-06

**Authors:** Yanshuo Pan, Binhui Liu, Wenying Zhang, Shan Zhuang, Hongzhe Wang, Jieyin Chen, Liang Xiao, Yuzhong Li, Dongfei Han

**Affiliations:** 1 School of Environmental Science and Engineering, Suzhou University of Science and Technology, Suzhou, China; 2 State Key Laboratory of Efficient Utilization of Arid and Semi-arid Arable Land, Beijing, China; 3 Institute of Environment and Sustainable Development in Agriculture, Chinese Academy of Agricultural Sciences, Beijing, China; 4 College of Natural Resources and Environment, Northwest A&F University, Yangling, Shaanxi, China; 5 Key Laboratory of Crop Drought Resistance Research of Hebei Province/Institute of Dryland Farming, Hebei Academy of Agriculture and Forestry Sciences, Hengshui, Hebei, China; 6 State Key Laboratory for Biology of Plant Diseases and Insect Pests, Institute of Plant Protection, Chinese Academy of Agricultural Sciences, Beijing, China; 7 Western Agricultural Research Center, Chinese Academy of Agricultural Sciences, Changji, China; 8 BGI-Shenzhen, Shenzhen, China; 9 College of Life Sciences, University of Chinese Academy of Sciences, Beijing, China; 10 Qingdao-Europe Advanced Institute for Life Sciences, BGI-Shenzhen, Qingdao, China; 11 China National GeneBank, BGI-Shenzhen, Shenzhen, China; 12 Shenzhen Engineering Laboratory of Detection and Intervention of human intestinal microbiome, BGI-Shenzhen, Shenzhen, China; 13 BGI College & Henan Institute of Medical and Pharmaceutical Sciences, Zhengzhou University, Zhengzhou, China; University of Hawaii at Manoa, Honolulu, Hawaii, USA

**Keywords:** drought, wheat, fungal community assembly, generalists, specialists

## Abstract

Beneficial interactions between plants and rhizosphere fungi can enhance plant adaptability during drought stress. However, harnessing these interactions will require an in-depth understanding of the response of fungal community assembly to drought. Herein, by using different varieties of wheat plants, we analyzed the drought-induced changes in fungal community assembly in rhizosphere and bulk soil. We demonstrated that drought significantly altered the fungal communities, with the contribution of species richness to community beta diversity increased in both rhizosphere and bulk soil compartments during drought stress. The stochastic processes dominated fungal community assembly, but the relative importance of deterministic processes, mainly homogeneous selection, increased in the drought-stressed rhizosphere. Drought induced an increase in the relative abundance of generalists in the rhizosphere, as opposed to specialists, and the top 10 abundant taxa that enriched under drought conditions were predominantly generalists. Notably, the most abundant drought-enriched taxon in rhizosphere was a generalist, and the corresponding *Chaetomium* strain was found capable of improving root length and activating ABA signaling in wheat plants through culture-based experiment. Together, these findings provide evidence that host plants exert a strong influence on rhizospheric fungal community assembly during stress and suggest the fungal communities that have experienced drought have the potential to confer fitness advantages to the host plants.

**IMPORTANCE:**

We have presented a framework to integrate the shifts in community assembly processes with plant-soil feedback during drought stress. We found that environmental filtering and host plant selection exert influence on the rhizospheric fungal community assembly, and the re-assembled community has great potential to alleviate plant drought stress. Our study proposes that future research should incorporate ecology with plant, microbiome, and molecular approaches to effectively harness the rhizospheric microbiome for enhancing the resilience of crop production to drought.

## INTRODUCTION

Drought is the primary abiotic stress affecting plant growth, leading to substantial threats to global crop yields (1). Soil fungi are widely distributed in global terrestrial ecosystems and account for more biomass than bacteria, and many studies have shown that they have great potential to alleviate plant drought stress (2–4). Plant growth during drought stress can influence the assembly of fungal communities, with pronounced effects in the rhizosphere (5). As such, the utilization of beneficial plant-fungus interactions has emerged as a complementary approach to alleviate drought stress in crop plants (4), and a better understanding of the ecological principles governing fungal community assembly under stress is needed.

As is generally acknowledged, the processes that drive microbial community assembly can be grouped into two ecological processes, i.e., deterministic and stochastic processes (6). Deterministic process suggests that the species traits, interspecies interactions (e.g., competition, predation, mutualisms, and trade-offs), and environmental conditions (e.g., pH, temperature, and moisture) are deterministic factors that control community assembly (7). Conversely, neutral theory hypothesizes that community assembly is unaffected by species traits but is governed by stochastic processes like birth, death, colonization, extinction, and speciation (8). Studies have provided valuable insights into how eco-evolutionary processes govern the assemblage of rhizospheric microbial communities, revealing that the rhizospheric microbiota is primarily influenced by slowly changing environmental factors (9, 10). Therefore, the contribution of deterministic and stochastic processes to fungal community assembly may vary between well-watered and drought-stressed soils. Evaluating these processes can aid in determining the drought-mediated alterations in community assembly.

The survival strategies of microorganisms enable them to thrive in diverse environments, inhabit a wide range of ecological niches, and participate in soil processes across various habitats (11). Based on the niche breadth of microbial taxa, they can be categorized as generalists, specialists, and opportunists (intermediate environmental tolerances) (12). Investigating their habitat niche breadths and distribution can yield valuable insights into the adaptive capacities and responses of soil microorganisms to environmental changes (13). Microbial generalists and specialists impart different impacts on microbial community dynamics. Generalists demonstrate broad environmental tolerances, encompassing a wider range of habitats than specialists (14). In comparison, specialists exhibit narrow tolerances and restricted distributions, making them more susceptible to extinction during environmental stress (15). These suggest that habitat generalists are less affected or more effectively buffered against environmental stress compared to specialists. However, how they respond differently to drought stress in the rhizosphere fungal communities remains unclear.

Wheat (*Triticum aestivum* L.) is the major food crop worldwide (16), relying on adequate water supply for agricultural production (17). In the last 35 years, drought stress has reduced global yields of wheat cultivation by about 21% (1). The North China Plain is the largest winter wheat cultivation area in China, contributing to more than 50% of China's wheat production (18, 19). However, it is frequently hit by drought, and the growth of wheat relies on irrigation due to insufficient rainfall to meet crop water requirements (20). Hence, gaining an in-depth understanding of the effects of drought on plant-fungus interactions is essential, especially in drought-sensitive crops. Here, we conducted a field experiment in the North China Plain. To enhance the credibility and generalizability of the results, the experiment involved seven widely grown wheat varieties, each cultivated under both well-watered and drought-stressed conditions. The seven wheat varieties exhibited differential sensitivity to drought, and there was no correlation with their genetic distance. We hypothesized that (i) plant and drought stress would drive the assembly of rhizospheric fungal communities and thus exert greater deterministic selection on them and that (ii) generalists, not specialists, would enrich in drought-stressed rhizosphere and have great potential to improve plant growth. To test our hypotheses, we investigated the drought-induced changes in fungal community assembly in bulk and rhizosphere soils. Additionally, the impact of drought-enriched fungi on plant growth was assessed by isolating corresponding strain from the rhizosphere.

## MATERIALS AND METHODS

### Field experiment and sample collection

**Fig 1:**
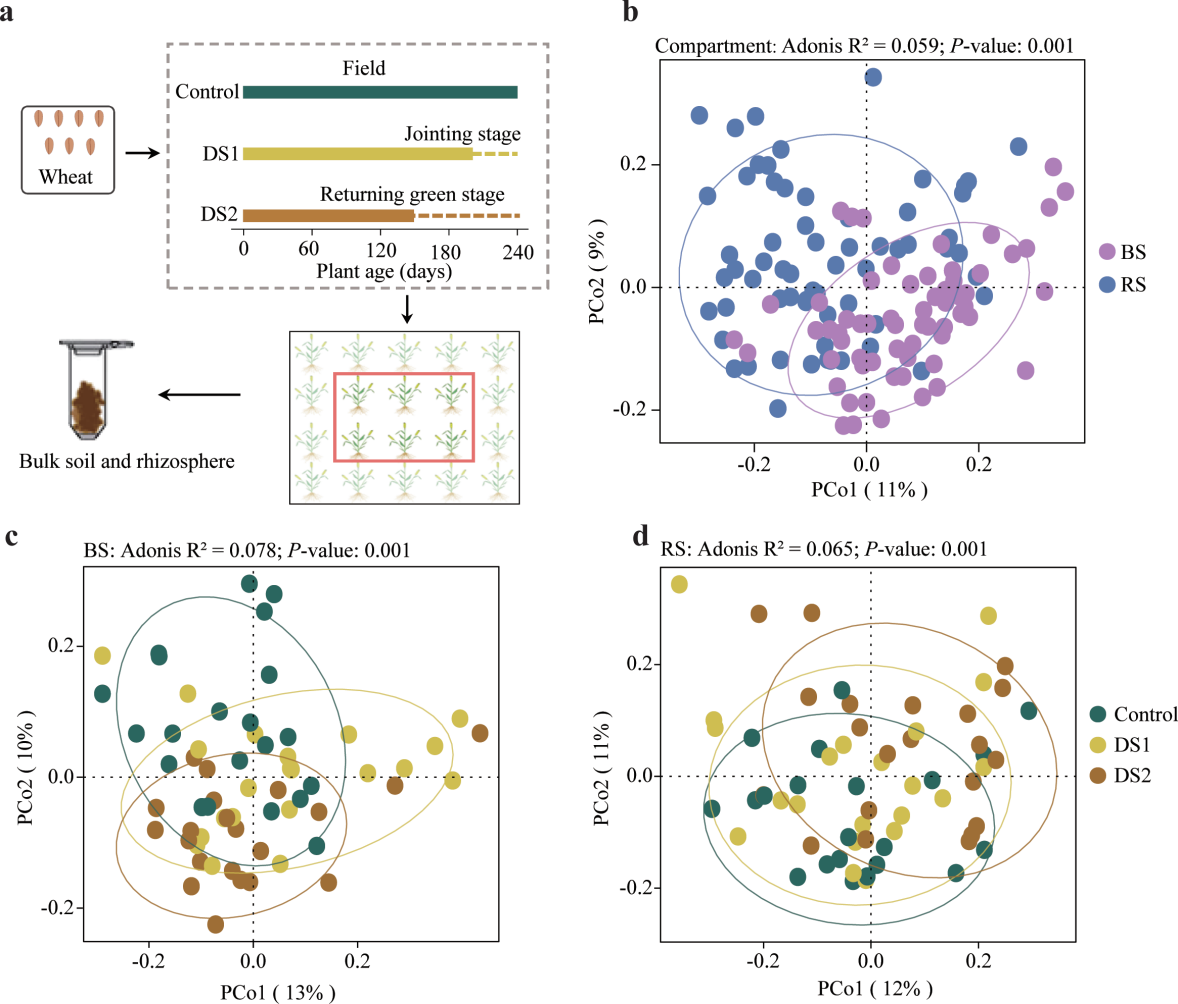
Variation of fungal communities in field conditions. (a) Diagram of the experimental design for wheat field trials. Solid and dotted lines are timeline of the watering regimes followed by control and drought-stressed (DS1 and DS2) wheat conditions; dotted line indicates no irrigation. Each harvested sample includes several individual wheat plants. (b–d) Unconstrained PCoA with Bray-Curtis distance showing that the fungal communities separate between bulk soil and rhizosphere (b) and among different drought treatments in bulk soil (c) and rhizosphere (d)(*P* < 0.001; permutational multivariate analysis of variance [PERMANOVA] by Adonis). Ellipses cover 85% of the data for each group.

The field experiment was conducted in the Hengshui city of North China Plain (37°60′N, 116°02′E), an area with a long history of wheat-maize crop rotation, and an annual mean temperature of 13.9℃, along with an average precipitation of 493 mm. The experiment consisted of three replications of seven wheat varieties subjected to well-watered (Control) and two drought-stressed (DS1 and DS2) conditions, employing a completely randomized block design (each plot was 60 m^2^). The treatments were designed as follows: (i) Control, full irrigation during the whole growth stage; (ii) DS1, stopping irrigation at jointing stage; and (iii) DS2, stopping irrigation at returning green stage (Fig. 1a). Furthermore, both drought treatments of DS1 and DS2 significantly reduced wheat yield compared to the control treatment (Table S1).

Both bulk soil and rhizosphere samples were collected during the filling stage in May 2020. Topsoil samples (0–20 cm) were collected using an auger as bulk soil, ensuring a distance of at least 20 cm from wheat plants. Rhizosphere samples were obtained by randomly extracting 10 wheat individuals in each plot and gently shaking off the soil loosely adhering to roots. Subsequently, the soil tightly adhered to roots was thoroughly washed with sterile H_2_O. After centrifugation at 3,000 × g for 5 min, the supernatants were discarded, and the pellets were rhizosphere samples. All collected samples were stored at −20℃ for subsequent analysis.

### DNA extraction and high-throughput sequencing

Soil DNA was extracted from 0.5 g of fresh soil using the FastDNA SPIN Kit for Soil (MP Biomedicals, Santa Ana, CA) following manufacturer's instructions, and the extracted DNA was stored at −20°C. The fungal internal transcribed spacer 2 region (ITS2) was amplified using PCR by target primer pairs gITS7 (5′-GTGARTCATCGARTCTTTG-3′)/ITS4 (5′-TCCTCCGCTTATTGATATGC-3′) (21). Each sample was amplified in triplicate within a 25 µL reaction mixture, including 12.5 µL of Premix Taq Version 2.0 (Takara Biotechnology), 0.5 µL of barcoded forward and reverse primers (10 µM), 1 µL of diluted DNA, and 10.5 µL of sterile H_2_O. After an initial denaturation step at 94°C for 5 min, the targeted region was amplified through 35 cycles of 94°C for 30 s, 56.5°C for 30 s, and 72°C for 30 s, followed by a final elongation step of 7 min at 72°C. The triplicate PCR products for each sample were verified using 1.2% agarose gel electrophoresis and then pooled, followed by purification using the EZNA Cycle-Pure Kit (Omega Bio-tek Inc., Doraville, GA), respectively. Finally, the purified amplicons were pooled by normalizing in equimolar numbers and sequenced using the MGISEQ-2000 platform (BGI, China).

Raw sequences were analyzed using the Quantitative Insight into Microbial Ecology (QIIME 2, v2020.2) pipeline (https://view.qiime2.org). Briefly, the primers were removed by cutadapt (v2018.11.0) (22) and submitted to QIIME2 as paired-end reads. Sequences were quality controlled, chimera filtered, denoised, and merged using DADA2 plugin by running “qiime dada2 denoise-paired” command (23). Subsequently, they were clustered into operational taxonomic units (OTUs) with a 97% similarity threshold using the “qiime vsearch cluster-features-de-novo” command. OTUs were assigned taxonomy using UNITE database (https://unite.ut.ee/) through the “qiime feature-classifier fit-classifier-naive-bayes” command. For alpha (α) and beta (β) diversity analysis, all samples were rarefied to 14,000 sequences through the “qiime diversity core-metrics” command.

### Bioinformatic analysis

Non-metric multidimensional scaling (NMDS) and pairwise permutational analysis of variance (PERMANOVA) of fungal communities were performed based on Bray-Curtis distance through the vegan package (24) in R v.3.6.1 (25). Significant discriminating fungal taxa (*P* < 0.05) were determined by R package edgeR (26) and visualized using R package ggtern and GraPhlAn software (27, 28). Community dissimilarity (beta diversity; BDtotal) was divided into species richness difference (RichDif) and replacement (Repl) components using the adespatial package (29). The stochasticity in fungal community assembly was assessed using the beta nearest taxon index (βNTI) (30, 31). As the βNTI analysis required a reliable phylogenetic tree to measure the average nearest taxon relationships, fungal ITS2 region might not be utilized as the highly divergent marker (32). Thus, we constructed the fungal phylogenetic tree relied on a fungal phylogeny based on 18S rRNA + 28S rRNA gene sequences (taxonomy_to_tree.pl) (33). To calculate the βNTI, β-mean-nearest taxon distances (βMNTD) were first analyzed by R package picante (34), then the βMNTD null model was generated by randomly shuffling the tips of the phylogenetic tree and repeated 999 times. The value of |βNTI| > 2 means that community assembly is governed primarily by deterministic processes, while the value of |βNTI| < 2 indicates that weak selection pressure and community assembly are likely dominated by stochastic processes (35). Moreover, the deterministic and stochastic processes were categorized into five ecological processes based on both βNTI and Bray-Curtis-based Raup-Crick Index (RCBray) values, including heterogeneous selection (βNTI < −2), variable selection (βNTI > 2), dispersal limitation (|βNTI| < 2 and RCBray > 0.95), homogenizing dispersal (|βNTI| < 2 and RCBray < −0.95), and undominated (|βNTI| < 2 and |RCBray| < 0.95) (36, 37). Habitat niche breadth was quantified by Levins' niche breadth index (B) equation, and the community-level B value (Bcom) was calculated as the average of B values from all species present within a community (12). A high B value suggests that the OTU is distributed extensively and uniformly across a broad spectrum of locations, indicating a broad habitat niche breadth. The community-level B value (Bcom) was calculated as the mean of B values from all taxa present within a given community. To identify the generalists, specialists, and opportunists, the occurrences of OTUs were derived by simulating 1,000 permutations using the R package EcolUtils (38). Specialists are defined by observed occurrences of niche breadth falling below the lower 95% confidence interval (CI), while generalists exceed the upper 95% CI, and OTUs with observed occurrences within the 95% CI are opportunists (39).

### Fungal strain isolation and identification

Isolation of fungal strains was carried out using the drought-stressed rhizosphere soil collected from the field. Briefly, 1 g of rhizosphere soil was mixed with 9 mL of sterile water in a rotary shaker (180 rpm) at 28°C for 30 min, followed by serial dilution and plating onto solid Rose bengal agar (RBA) and incubated at 28°C in dark. Fungal colonies were then purified by streaking on potato dextrose agar (PDA) plates. A total of 16 purified strains were obtained and stored in 25% glycerol at −80°C (Table S2). The resulting strains were amplified by PCR using the primer pair of ITS1 (5′-TCCGTAGGTGAACCTGCGG-3′)/ITS4 (5′-TCCTCCGCTTATTGATATGC-3′) and sequenced by Sanger sequencing (Allwegene, Beijing, China). Sequences were quality checked, trimmed, and BLAST searched against the nucleotide database of NCBI. Finally, an isolate named *Chaetomium* sp. DR413 was used for subsequent laboratory experiment.

### Laboratory experiment to assess the effect of *Chaetomium* sp. DR413 on wheat growth

A laboratory experiment was built to assess the impact of isolate *Chaetomium* sp. DR413 (DR413) on wheat growth. All seven wheat varieties were included in the experiment and cultivated in see-through tubes (5 cm diameter × 40 cm height). The soils that were previously collected from North China Plain were γ-sterilized, and each see-through tube contained 100 g of sterilized soil. To obtain spore suspensions, the strain DR413 was grown on PDA plate at 28°C for 7 days, and then the spores were harvested and suspended in sterile water and counted using a hemocytometer (40). Wheat seeds were surface-sterilized by immersing them in 75% ethanol for 30 s, then in 2.5% sodium hypochlorite three times for 15 min, and finally washed extensively with sterile water (41). Surface-sterilized seeds were placed on Petri dishes with moist gauze, and they were divided into two groups. One group was assigned as the non-inoculated group, treated only with sterile water. Another group was inoculated with spore suspensions of DR413 (10^8^ spores ml^−1^) as inoculated group (42). All dishes were incubated at 28°C for 48 h for germinating. After germination, each see-through tube was planted with germinated seeds. Soil moisture was adjusted to 80% and 50% of water holding capacity (WHC) for the well-watered (Control) and drought-stressed (DS) treatments, respectively. Finally, all tubes were sealed using sterile breathable sealing film and placed in a growth chamber under 16 h of light at 20°C/8 h of dark at 15°C, a relative humidity of 65% (light)/72% (dark), and a photosynthetically active radiation of 500 µmol m^−2^ s^−1^. Altogether, the experiment included three replicates of seven wheat varieties, two inoculation types (with or without inoculation of DR413), and two water conditions (Control, DS), resulting in a total of 84 samples. Following a 3-week incubation period, measurements were taken for wheat shoot height, root length, and fresh weight.

### RNA extraction and RT-qPCR of ABA signaling genes

Plant RNA was extracted from three (H6632, HN6119, and ZXM99) out of seven wheat varieties, using the fresh roots by the EZNA Plant RNA kit (Omega Bio-tek, USA). The quantities, purities, and integrities of the extracted RNA were evaluated by agarose gel electrophoresis and NanoDrop spectrophotometer. Complementary DNA (cDNA) was synthesized from 1 µg of RNA in a 20 µL reaction mixture using the anchored oligo(dT) primers provided by EasyScript One-Step gDNA Removal and cDNA Synthesis SuperMix kit (Transgen Biotech, China). The expressions of marker genes for abscisic acid (ABA) signaling pathway were quantified by quantitative real-time polymerase chain reaction (RT-qPCR) using Taq Pro Universal SYBR qPCR Master Mix (Vazyme Biotech Co., Ltd, Najing, China) on an ABI 7500 Real Time PCR system (Applied Biosystems, Foster City). A total of 10 genes were analyzed, comprising marker genes encoding mitogen-activated protein kinases (MAPKs: *TaMAPK3*, *TaMAPK12;1,* and *TaMAPK16*), transcription factors (TFs: *TaNAC2*, *TaSIM*, and *TabZIP15*), protein phosphatase 2Cs (PP2Cs: *TaPP2C-a7*, *TaPP2C-a10,* and *TaPP2C-a30*), and sucrose non-ferment 1-related protein kinase 2 (SnRK2: *TaSnRK2.9*) (43–45). The *TaGAPDH* gene was utilized as a housekeeping gene for normalization. Gene expressions were quantified using the 2^−ΔΔCT^ (cycle threshold) method (46). A detailed information about the primers and thermal conditions was listed in Table S3.

## RESULTS

### Composition and diversity of the fungal communities

After sequencing, a total of 10,527,843 high-quality sequences were obtained (with an average of 83,554 and ranging from 15,636 to 179,923). At phylum level, the fungal communities were predominated by Ascomycota, Basidiomycota, Chytridiomycota, Mortierellomycota, and Mucoromycota, across all samples of bulk soil and rhizosphere (Fig. S1). During drought stress, the proportions of Ascomycota (Control: 58.41%, DS1: 65.56%, and DS2: 65.89%) increased in bulk soil, while the Chytridiomycota (Control: 4.45%, DS1: 5.00%, and DS2: 9.10%) and Mucoromycota (Control: 4.67%, DS1: 9.37%, and DS2: 5.89%) showed an increase in rhizosphere. In comparison to control treatment, the Shannon index of bulk soil significantly decreased in DS2 treatment (*P* < 0.05), while rhizospheric mycobiomes exhibited no significant changes during drought stress (Fig. S2). The NMDS and ADONIS analysis revealed that the fungal communities were clearly segregated by soil compartments (R^2^ = 0.059; *P* < 0.001) and wheat varieties (R^2^ = 0.139; *P* < 0.001) (Fig. 1b; Fig. S3). Additionally, a significant influence of drought on the fungal communities was observed in both bulk soil (R^2^ = 0.078: *P* < 0.001) and rhizosphere (R^2^ = 0.065; *P* < 0.001) (Fig. 1c and d). In every variety, the fungal communities were significantly impacted by drought and soil compartments (*P* < 0.001) (Fig. S4).

**Fig 2:**
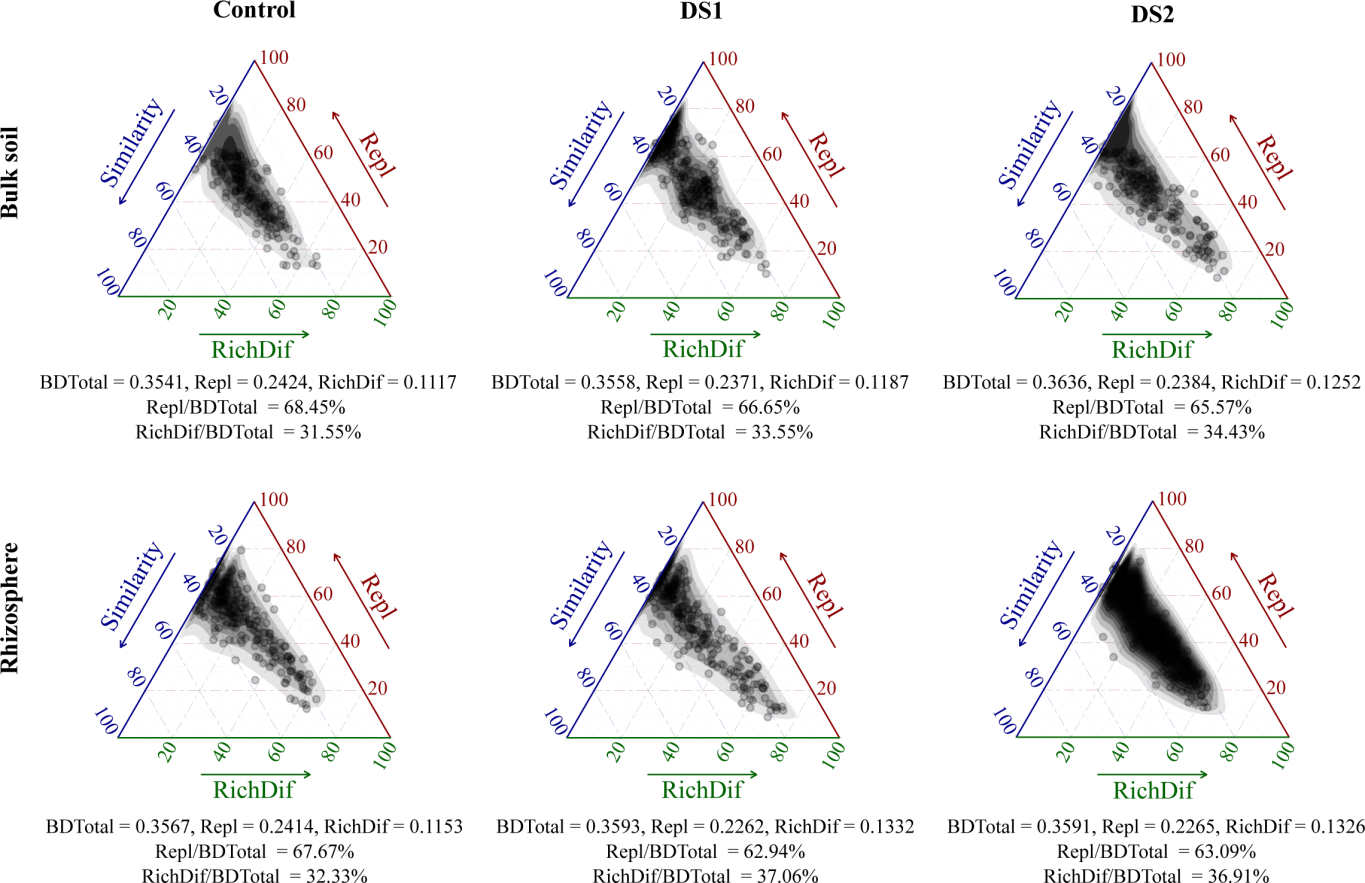
Ternary plots of beta diversity components for fungal taxa. bdtotal (dissimilarity) is the microbial beta diversity, Repl is the total replacement diversity, and RichDif is the total richness difference (species richness) diversity. BDtotal = RichDif + Repl and Similarity = 1 − Bdtotal. Repl/BDTotal is the relative contribution of Repl to microbial beta diversity, and RichDif/BDTotal is the relative contribution of RichDif to microbial beta diversity.

The beta diversity decomposition analyses demonstrated that fungal community compositional dissimilarities (BDtotal) were dominated by species replacement processes (Repl/BDtotal) (62.94–68.45%), rather than richness difference processes (RichDif/BDtotal) (31.55–37.06%), in the soil compartments (Fig. 2). However, drought decreased the contribution of species replacement processes to fungal community compositional dissimilarities, but it increased the contribution of richness difference processes in both bulk soil (Control: 31.55%, DS1: 33.55%, and DS2: 34.43%) and rhizosphere (Control: 32.33%, DS1: 37.06%, and DS2: 36.91%). These results indicated that, while species replacement processes were the primary contributors to fungal community compositional dissimilarities, drought enhanced the contribution of richness difference processes.

### Assembly of the fungal communities

**Fig 3:**
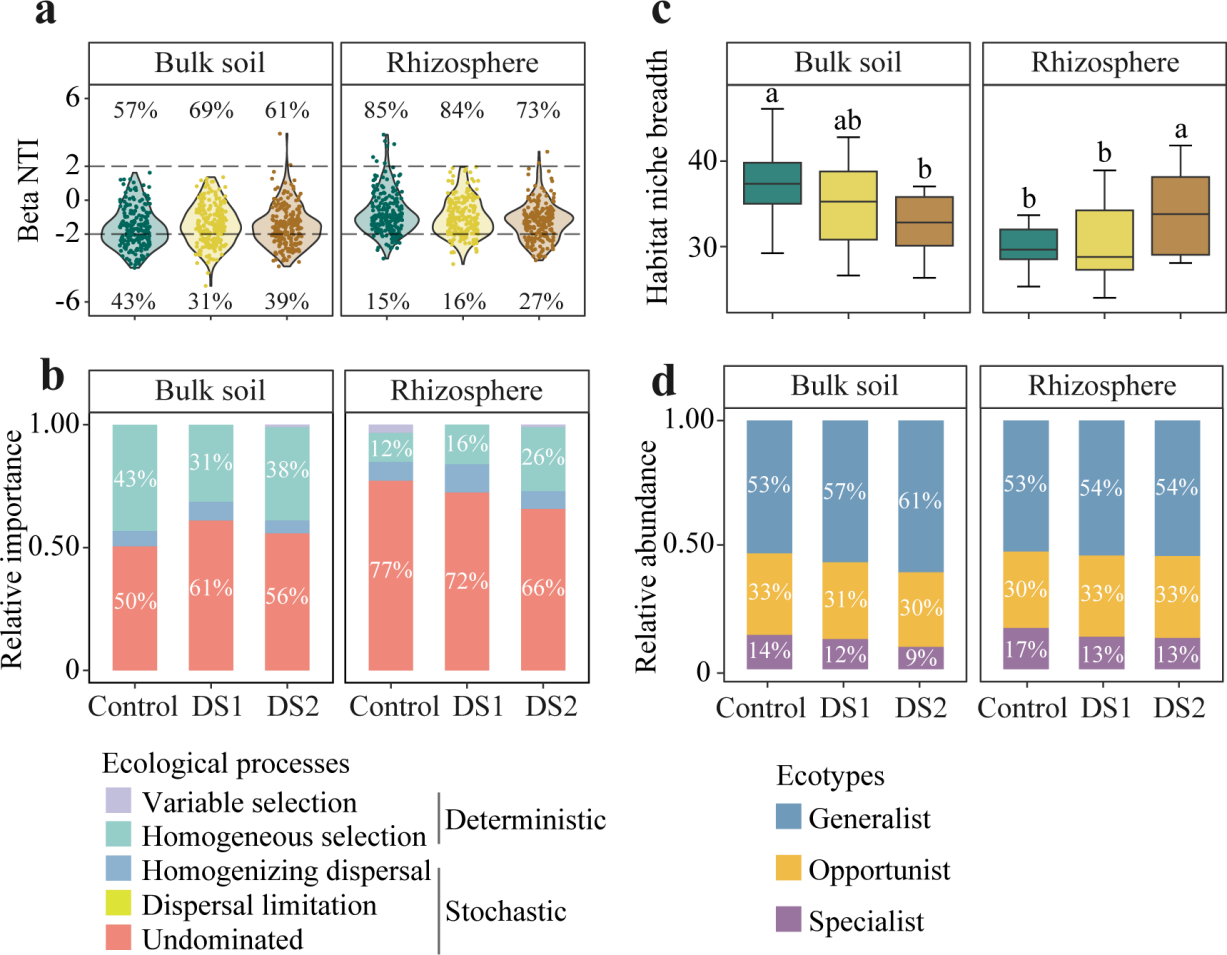
Fungal community assembly processes in bulk soil and rhizosphere. (a) Habitat niche breadth of bacterial communities. (b) Relative contribution of determinism and stochasticity on mycobiome assembly based on the β-nearest taxon index (βNTI) values. The |βNTI| < 2 and |βNTI| ≥ 2 represent dominant stochasticity and determinism in driving mycobiome assembly, respectively. The percentages above and below the violin plot represent the proportion of the stochastic processes and deterministic processes in microbiome assembly, respectively. (c) Relative abundance of generalists, opportunists, and specialists in the total fungal community composition. (d) Relative importance of five ecological processes (heterogeneous selection: βNTI < −2, variable selection: βNTI > 2, dispersal limitation: |βNTI| < 2 and RCBray > 0.95, homogenizing dispersal: |βNTI| < 2 and RCBray < −0.95, and undominated: |βNTI| < 2 and |RCBray| < 0.95) in microbiome assembly based on the β-nearest taxon index (βNTI) and Bray-Curtis-based Raup-Crick Index (RCBray).

Null model analysis was used to investigate the impact of drought stress on the relative contribution of deterministic (|βNTI| ≥ 2) and stochastic (|βNTI| < 2) processes in mycobiome assembly (Fig. 3a and b). In bulk soil, drought increased the contribution of stochastic processes to community assembly (Fig. 3a). On the contrary, the contribution of deterministic processes to community assembly increased in drought-stressed rhizosphere. Drought-induced changes in the deterministic process of homogeneous selection were responsible primarily for turnover of the fungal community assembly. The relative importance of homogeneous selection in community assembly decreased in bulk soil but increased in rhizosphere under drought conditions (Fig. 3b), indicating that the impact of drought on rhizospheric community assembly is influenced by host plants.

To explore the metabolic flexibility of fungal communities during drought stress, the mean community-level habitat niche breadth was analyzed in both bulk soil and rhizosphere (Fig. 3c). The habitat niche breadth values of soil fungal communities exhibited contrasting decreasing and increasing trends with increased drought stress in bulk soil and rhizosphere, respectively. By comparing the habitat niche breadth, the OTUs were defined as generalists, opportunists, and specialists. In total, there were 98 OTUs identified as generalists, 321 OTUs identified as opportunists, and 299 OTUs identified as specialists (Fig. S5). While the number of generalists were approximately one-third that of specialists, generalists exhibited nearly 4–5 times higher relative abundance than specialists in each treatment (Fig. 3d). In addition, drought-induced increase in the relative abundance of generalists and decrease in the relative abundance of specialists were observed in both bulk soil and rhizosphere.

### Differential abundance of fungal taxa during drought stress

**Fig 4:**
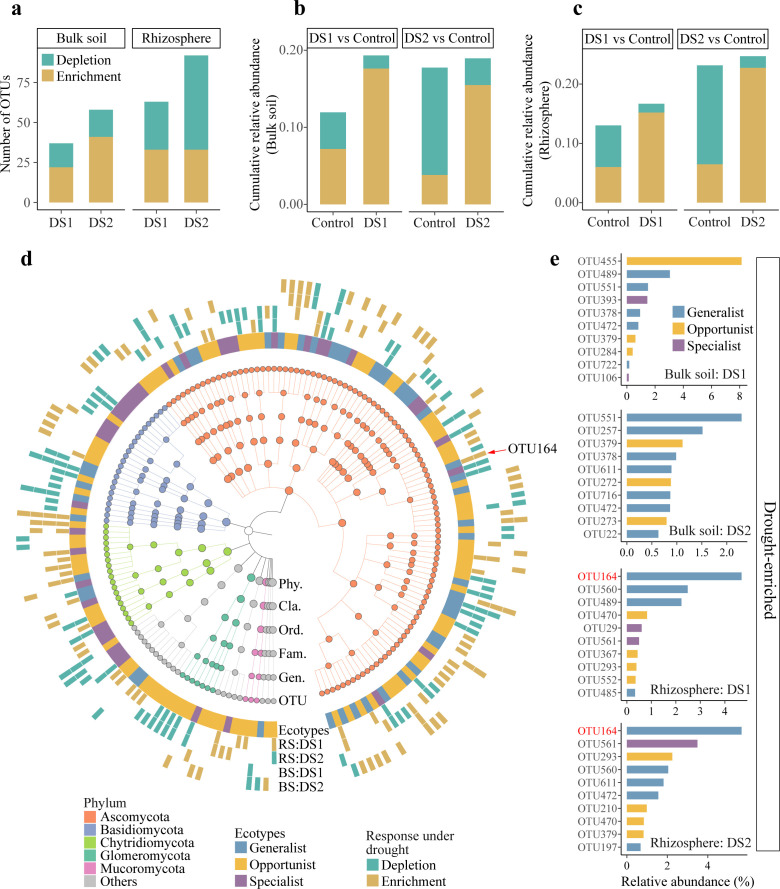
Drought-responsive OTUs in bulk soil and rhizosphere. (a) Number of OTUs detected as differentially abundant (*P* < 0.05) between control and drought-stressed (DS1 and DS2) samples. (b and c) Cumulative relative abundances of drought-responsive OTUs in bulk soil (b) and rhizosphere (c). (d) Taxonomy dendrogram displaying the drought-responsive OTUs detected across all drought-stressed soils. The inner ring indicates the ecotypes (generalist: blue, opportunist: orange, and specialist: purple) of the drought-responsive OTUs, and the four outermost rings indicate that an OTU was significantly higher (brown) or lower (green) under drought in the rhizosphere (RS) and bulk soil (BS) communities. The nodes in the cladogram indicate the phylum (Phy.), class (Cla.), order (Ord.), family (Fam.), and genus (Gen.) to which each OTU belongs. (e) Relative abundance of the 10 most abundant OTUs that were enriched in bulk soil and rhizosphere under drought stress. The colored bar indicates the ecotype to which the taxon belongs.

To identify taxa that were enriched or depleted in drought-stressed communities, the differential abundance patterns of OTUs were compared between control and drought (DS1 and DS2) treatments. A total of 177 drought-responsive OTUs were detected, with a higher number of differentially abundant OTUs found in the rhizosphere (DS1: 63; DS2: 92) than in bulk soil (DS1: 37; DS2: 58) (Fig. 4a; Table S4). In bulk soil, there was a little difference in the cumulative relative abundance of drought-responsive OTUs detected in DS1 (19.32%) and DS2 (18.94%) treatments (Fig. 4b). Nevertheless, the drought-responsive OTUs detected in DS2 (24.70%) treatment accounted for higher cumulative relative abundance compared to DS1 (16.68%) treatment in rhizosphere (Fig. 4c). Additionally, among the drought-responsive OTUs, 47 were generalists, 93 were opportunists, and 39 were specialists (Fig. 4d). In DS1 and DS2 treatments, 6 and 14 generalists, along with 4 and 4 specialists, were significantly enriched in the drought-stressed bulk soil; while 8 and 9 generalists, as well as 4 and 6 specialists, respectively, were significantly enriched in drought-stressed rhizosphere (Fig. 4d). Moreover, the top 10 most abundant drought-enriched OTUs in each treatment were mainly belonging to generalist (Fig. 4e). In particular, the generalist OTU164 was the most abundant taxon significantly enriched in both DS1 and DS2 treatments of rhizosphere during drought stress.

### Beneficial effects of *Chaetomium* sp. DR413 on wheat growth

**Fig 5:**
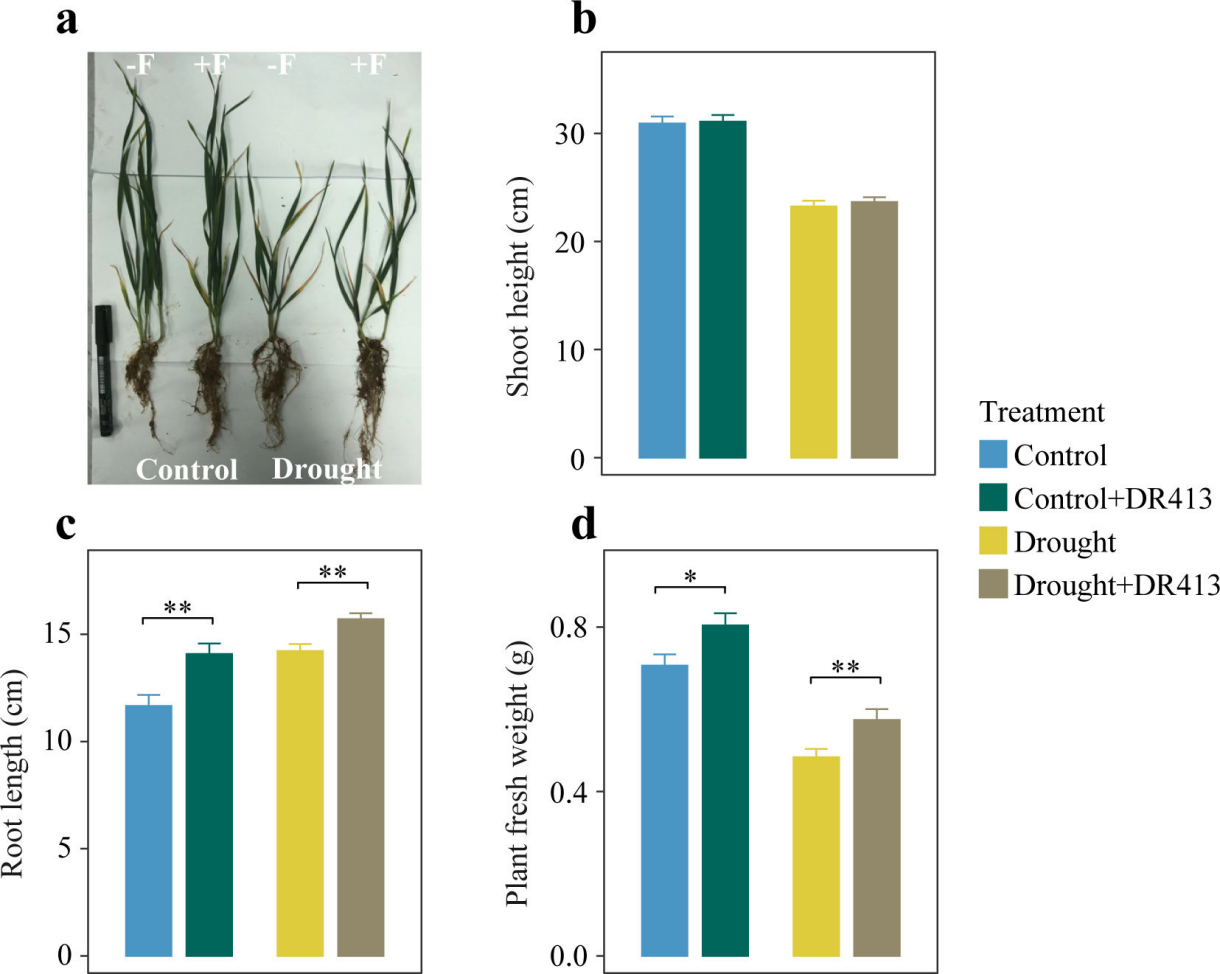
*Chaetomium* sp. DR413 (corresponding isolate of OTU164) significantly increases wheat root length and fresh weight in control and drought treatments. (a) Picture showing the harvested wheat plants grown in control and drought-stressed soils with (+F) or without (−F) inoculation of DR413. (b–d) Effects of DR413 inoculation on wheat shoot height (b), root length (c), and fresh weight (d) in control and drought-stressed treatments. Asterisks indicate significant differences between treatments (*P < 0.05 and **P < 0.01). Error bars in panels b–d represent standard errors of the mean (*n* = 21).

To evaluate the influence of OTU164 on plant growth, the corresponding strain was screened from the same rhizosphere soil previously collected from field (Fig. S6). An isolate demonstrating 100% nucleotide similarity in ITS gene with the sequence of OTU164 was identified as the corresponding strain of OTU164 (Fig. S7) and named *Chaetomium* sp. DR413 (DR413). Based on this, a laboratory-based experiment was built to determine the ability of OTU164 to enhance plant growth using the corresponding strain DR413 (Fig. S6). Obviously, drought severely inhibited the growth of wheat, but the inoculation with DR413 could mitigate the adverse stress of wheat induced by drought in five out of seven wheat varieties (Fig. 5a; Fig. S9). For drought-stressed samples, the inoculation of DR413 did not affect shoot height of wheat plants but significantly increased the root length (+110%) and plant fresh weight (+119%) (Fig. 5b through d). Meanwhile, the beneficial effects of DR413 on wheat growth were also observed in control treatment, leading to increased root length (+122%) and plant fresh weight (+128%).

**Fig 6:**
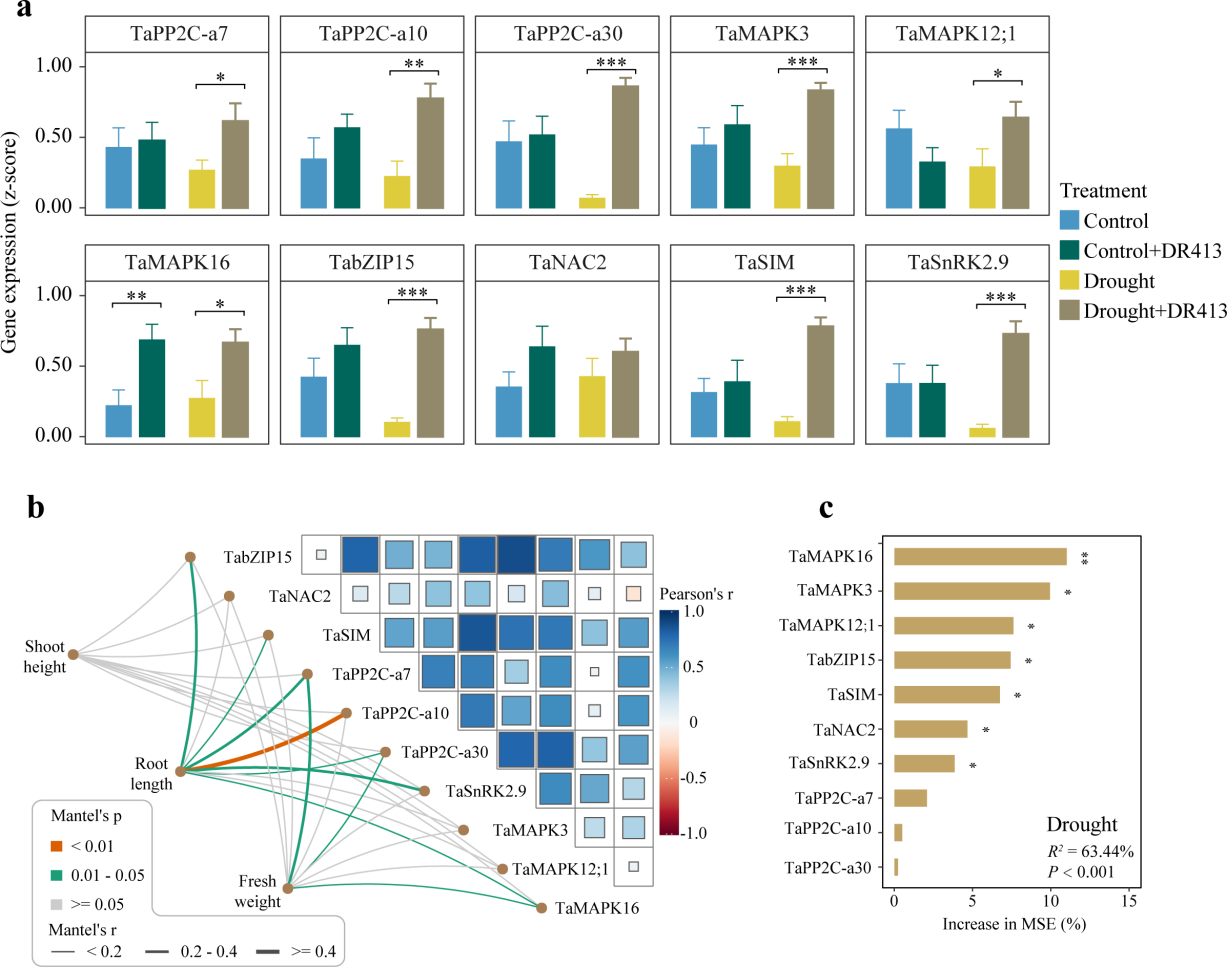
*Chaetomium* sp. DR413 significantly activates ABA signaling in wheat cultivars grown in drought-stressed soil. (a) Effects of DR413 inoculation on the transcription of genes associated with abscisic acid (ABA) signaling pathway. Error bars represent standard errors of the mean (*n* = 9). (b) Mantel’s correlations between wheat shoot height, root length, and fresh weight with ABA signaling pathway genes under drought stress. Edge width corresponds to the Mantel’s r statistic, and edge color denotes the statistical significance. (c) Contribution of ABA signaling pathway genes in predicting wheat root length under drought stress based on random forest modeling analyses. *P < 0.05, **P < 0.01, and ***P < 0.001. MSE, mean square error.

The plant growth-promoting ability of DR413 was further assessed by investigating the expression of ABA signaling genes in three wheat varieties (H6632, HN6119, and ZXM99). The inoculation of DR413 stimulated root elongation specifically under drought conditions in these varieties, while exhibiting no influence on root length under well-watered conditions. A group of crucial marker genes for ABA signaling was quantified, including those encoding protein phosphatase 2Cs (PP2Cs), sucrose non-fermenting 1-related protein kinase 2 (SnRK2), mitogen-activated protein kinases (MAPKs), and transcription factors (TFs: TaNAC2, TaSIM, and TabZIP15) (Fig. S10). Except for the *TaMAPK16* gene, DR413 did not affect the expression of ABA signaling genes in control treatment (Fig. 6a). However, under drought conditions, the expression of ABA signaling genes, including *TaPP2C-a7*, *TaPP2C-a10*, *TaPP2C-a30*, *TaMAPK3*, *TaMAPK12;1*, *TaMAPK16*, *TabZIP15*, *TaNAC2*, *TaSIM*, and *TaSnRK2.9*, was all significantly enhanced by DR413 inoculation. The correlations between wheat shoot height, root length, and fresh weight with ABA signaling genes were further analyzed. In control treatment, no gene was significantly correlated with wheat root length, and the random forest modeling analysis indicated that the contribution of these marker genes to plant root length was not significant (14.4%; *P* = 0.112) (Fig. S11). In contrast, the root length of drought-stressed plants displayed significant correlations with most marker genes, including *TabZIP15*, *TaSIM*, *TaPP2C-a7*, *TaPP2C-a10*, *TaPP2C-a30*, *TaSnRK2.9,* and *TaMAPK16* (Fig. 6b). Moreover, these ABA signaling genes significantly contributed to the variation in plant root length (64.3%; *P* < 0.001) (Fig. 6c), suggesting their crucial roles in the growth of drought-stressed plants.

## DISCUSSION

### Variations in the composition of fungal communities

Rhizosphere is a unique ecological niche with tight plant-microorganism interaction; in adverse conditions, changes in the rhizospheric mycobiomes are the result of the combined effects of biotic/abiotic stressors and host plants (47). Consistent with a previous study (5), we observed that the host plants and drought stress were responsible for the changes in rhizospheric fungal communities. In rhizosphere, plants can mediate interactions with soil microorganisms via root exudation, either by promoting or inhibiting the growth of specific microbial taxa (48). It has been shown that drought-induced changes in exudate composition can in turn alter the activity of root-associated microorganisms (49). Additionally, drought may also potentially affect microbial community composition by changing the growth and morphology of roots (50). Moreover, we partitioned the community compositional dissimilarities into species replacement and richness difference processes, which can aid in a more comprehensive understanding of the processes contributing to assembly of microbial communities (51). Previous studies have reported that species replacement dominate in microbial communities in many ecological niches, a trend in line with our results, suggesting that microbial communities shared their species in a limited way (29, 52, 53). However, we found that drought reduced the contribution of species replacement but increased the contribution of richness difference to fungal communities. The species-energy theory posits that species richness is positively related to available energy (54). This suggests that the drought-induced decrease in soil nutrient availability can impact species richness, thereby leading to shift in the microbial communities from species replacement to richness difference, ultimately reducing the pool of microbial species.

### Drought increases the contribution of deterministic process to rhizospheric fungal community assembly

Stochastic and deterministic processes represent two complementary mechanisms of ecological forces that structure community assembly (32). We found the fungal community assembly was mostly regulated by undominated processes, which are processes induced by weak selection/dispersal, diversification, and drift (35). This aligns with prior researches suggesting that communities, such as fungi, characterized by low alpha diversity and richness are primarily mediated by undominated processes in the presence of weak environmental selection (55, 56). As a selection pressure on community assembly, drought stress imposes a strong and consistent environmental determinant leading to increased proportion of deterministic processes in community assembly (57). However, we found that drought increased the contribution of stochastic processes to the fungal community assembly in bulk soil. The unforeseeable spatiotemporal variations in environmental conditions, described as “environmental stochasticity”, are able to amplify uncertainties and fluctuations in microbial population and life history strategy (58). The previous study reported that intermediate disturbance (intermediate duration of dry period) can promote microbial stochastic processes (59), and this could be related with why drought induced a higher stochastic process in our study. Moreover, the response of fungi to drought stress was compartment-specific, as drought enhanced the contribution of deterministic processes to rhizospheric community assembly, indicating that the selection force of host plants on community assembly was enhanced under drought conditions. In detail, we found that the increased deterministic processes were attributed to the homogeneous selection. Homogeneous selection leads to low compositional turnover, making the communities be more phylogenetically similar (6). Therefore, the increased proportion of homogeneous selection in community assembly indicates that drought can lead to similar community structures in rhizosphere.

### Habitat generalists possess broader adaptations to drought than specialists

Ecological theory reveals that the distributions of habitat generalists and specialists would be differentially affected by habitat disturbance (60). The growth of specialists is hindered under adverse conditions; in contrast, generalists are expected to sustain more stable populations due to their greater adaptability to environmental change. A previous study revealed that environmental disturbance seemed to favor the growth of generalists (60). Our study demonstrated similar results: we found that drought increased the relative abundance of generalists rather than specialists in both bulk soil and rhizosphere, revealing that generalists exhibited greater competitiveness than specialists under drought conditions. The observation could be because the high metabolic flexibility of generalists confers them an advantage in better adapting to the drought-induced decrease in nutrient availability and shift in plant root exudates. Most notably, the 10 most abundant drought-enriched OTUs were mainly generalists in the two soil compartments. These findings hold crucial consequences for our understanding and modeling of biogeochemical processes, as the organism-centric approach suggests that the occurrence of a specific organism can dictate the ongoing process (61). Therefore, in disturbed conditions, dissecting the functional and metabolic characteristics of the stress-responsive generalists may be beneficial for predicting ecosystem processes.

### Drought-induced recruitment of rhizosphere generalists capable of enhancing plant adaptability to drought stress

The fundamental question is whether the observed drought-induced shift in the rhizospheric fungal community assembly can confer benefits to the host plants, especially in coping with drought stress. It has reported that soil microbial communities that have experienced drought together with plants can enhance their host fitness during future stress (62). Indeed, our culture-dependent work confirmed the plant growth-promoting ability of the most abundant rhizosphere fungus (OTU164). The OTU164 is a generalist, belonging to the genus *Chaetomium*. Colonization by *Chaetomium* sp. DR413, an isolate corresponding to OTU164, significantly increased root length and fresh weight in both well-watered and drought-stressed wheat seedlings. Root elongation can help plants cope with drought stress by enabling them to penetrate through hardpan soil, thereby improving their access to water and nutrients during periods of drought stress (63, 64). Moreover, multiple *Chaetomium* species have been shown to promote drought resistance in various plants, including maize (65), wheat (66), and rice (67), suggesting the potential beneficial role of *Chaetomium* enrichment in rhizospheric mycobiomes might be more general.

Additionally, we also measured the activity of ABA signaling to further assess whether DR413 can enhance the drought resistance in plants. ABA signaling represents a highly conserved drought response pathway, playing a crucial role in responding to drought, including core components such as PP2C, SnRK2, and TF, and crosslinks with MAPK cascades (45). We found that DR413 induced up-regulation of numerous marker genes encoding these signaling components during drought stress, while there was no obvious change in well-watered plants. These results indicate that DR413 can activate the ABA signaling pathway but it requires drought stress to be triggered. Previous molecular-based studies have indicated that the overexpression of ABA signaling pathway genes can enhance root length during stress (43, 44, 68). Similarly, our results demonstrated a significant correlation between wheat root length and the majority of marker genes. Furthermore, a random forest approach revealed that these genes significantly explained the variation in root length under drought conditions. Hence, a mechanism for DR413 to promote root elongation may be attributed to its activation of ABA signaling. There results provide support for “cry for help” hypothesis, which suggests that plant hosts can recruit specific microbes that are able to alleviate the abiotic/biotic stress, showing that it likely also occurs during drought stress (69–71). Further metagenomic and comparative genomic studies are required to elucidate the molecular mechanisms responsible for these responses.

### Conclusion

In conclusion, our results provide support for the hypothesis that plants can lead to re-assembly of rhizospheric fungal communities that enhance stress adaptation in plants when they are challenged by drought. Specifically, drought increases the contribution of species richness to community beta diversity and the importance of homogeneous selection in rhizospheric community assembly. We demonstrate that the greater metabolic flexibility of the fungal community confers an advantage to habitat generalists over specialists in adapting to growth in drought-stressed soils. Most importantly, the most abundant drought-enriched generalist, the genus *Chaetomium*, is substantiated to improve plant drought tolerance. This work enhances our understanding of rhizospheric mycobiome assembly during drought stress, suggesting the potential strategies to harness fungal communities for conferring drought tolerance in crops.

## Data Availability

Raw sequence data were deposited in the Sequence Read Archive (SRA) at NCBI under accession number PRJNA945607. The sequence of the ITS gene of *Chaetomium* sp. DR413 was deposited in the NCBI database under GenBank accession number OR770489. Additional data related to this paper may be requested from us.

## SUPPLEMENTAL MATERIAL

Supplemental figures and tables msystems.00354-24-s0001.xlsx Fig. S1 to S11; Tables S1 and S3.

Supplemental tables msystems.00354-24-s0002.docx Tables S2 and S4.

ASM does not own the copyrights to Supplemental Material that may be linked to, or accessed through, an article. The authors have granted ASM a non-exclusive, world-wide license to publish the Supplemental Material files. Please contact the corresponding author directly for reuse.
