# Supplementary material for: Drought-induced assembly of rhizosphere mycobiomes shows beneficial effects on plant growth
Source: mSystems. 2024 Jun 6;9(7):e00354-24. doi: 10.1128/msystems.00354-24 (PMC11264929; doi:10.1128/msystems.00354-24)
Supplement: Supplemental tables — Tables S2 and S4. [file msystems.00354-24-s0002.docx]

SUPPLEMENTARY INFORMATION

**Drought-induced assembly of rhizosphere mycobiomes** **shows beneficial effects on plant growth**

Yanshuo Pan^1,2,3,4†^ ˙ Binhui Liu^5†^ ˙ Wenying Zhang^5^ ˙ Shan Zhuang^3^ ˙ Hongzhe Wang^3^ ˙ Jieyin Chen^6,7^ ˙ Liang Xiao^8,9,10,11,12,13^ ˙ Yuzhong Li^3*^ ˙ Dongfei Han^1,2*^

^1^ School of Environmental Science and Engineering, Suzhou University of Science and Technology, Suzhou 215009, China

^2^ State Key Laboratory of Efficient Utilization of Arid and Semi-arid Arable Land in Northern China

^3^ Institute of Environment and Sustainable Development in Agriculture, Chinese Academy of Agricultural Sciences, Beijing 100081, China.

^4^ College of Natural Resources and Environment, Northwest A&F University, Yangling, Shaanxi 712100, China.

^5^ Key Laboratory of Crop Drought Resistance Research of Hebei Province / Institute of Dryland Farming, Hebei Academy of Agriculture and Forestry Sciences, Hengshui 053000, Hebei, China

^6^ State Key Laboratory for Biology of Plant Diseases and Insect Pests, Institute of Plant Protection, Chinese Academy of Agricultural Sciences, Beijing 100193, China

^7^ Western Agricultural Research Center, Chinese Academy of Agricultural Sciences, Changji 831100, China

^8^ BGI-Shenzhen, Shenzhen 518083, China

^9^ College of Life Sciences, University of Chinese Academy of Sciences, Beijing 100049，China

^10^ Qingdao-Europe Advanced Institute for Life Sciences, BGI-Shenzhen, Qingdao 266555, China

^11^ China National GeneBank, BGI-Shenzhen, Shenzhen 518120, China

^12^ Shenzhen Engineering Laboratory of Detection and Intervention of human intestinal microbiome, BGI-Shenzhen, Shenzhen 518120, China

^13^ BGI College & Henan Institute of Medical and Pharmaceutical Sciences, Zhengzhou University，Zhengzhou 450000，China

^†^ These authors contributed equally: Yanshuo Pan, Binhui Liu.

^*^ To whom correspondence may be addressed. Email: dongfeihan@usts.edu.cn, liyuzhong@caas.cn

Table S1 Drought effects on crop production of seven wheat varieties.

| **IDs** | **Full name** | **Wheat Yield**  **(kg/ha)** | | | **Wheat yield loss (%)** | | **Average wheat yield loss (%)** | **Resistance to drought stress** |
| --- | --- | --- | --- | --- | --- | --- | --- | --- |
|  |  | **Control** | **DS1** | **DS2** | **DS1** | **DS2** |  |  |
| H1401 | Heng 1401 | 6800(254)a | 6719(14)a | 5348(353)b | 1.18 | 21.35 | 11.27 | High |
| H6632 | Heng 6632 | 7824(185)a | 6920(327)b | 5117(226)c | 11.55 | 34.60 | 23.08 | Middle |
| HN6119 | Henong 6119 | 9444(250)a | 7546(408)b | 5929(212)c | 20.09 | 37.22 | 28.66 | Low |
| JinM47 | Jinmai 47 | 7315(311)a | 6563(11)b | 4808(34)c | 10.29 | 34.27 | 22.28 | Middle |
| JM325 | Jimai 325 | 9166(127)a | 8400(192)b | 5256(17)c | 8.36 | 42.65 | 25.51 | Low |
| YB700 | Yingbo 700 | 8484(29)a | 7444(76)b | 5772(789)c | 12.26 | 31.96 | 22.11 | Middle |
| ZXM99 | Zhongxinmai 99 | 7457(371)a | 5756(53)b | 5173(22)c | 22.82 | 30.63 | 26.73 | Low |

Data are means (±SD). Different letters within a column indicate differences at P < 0.05 (Tukey’s HSD test). Control: full irrigation; DS1, stopping irrigation at jointing stage; DS2, stopping irrigation at returning green stage. Wheat yield loss: (Control_yield_ – DS_yield_) / Control_yield_. Resistance to drought stress: High (Average wheat yield loss < 15%), middle (15% < Average wheat yield loss < 25%), and low (Average wheat yield loss > 25%).

Table S2 Taxonomy and sequences of the screened fungal strains.

Note: Table S2 is in another Supplemental Material.

Table S3 Primers and thermal conditions of RT-qPCR.

| Gene | Forward primer sequence  (5′-3′) | Reverse primer sequence  (5'-3') | Gene description | Tm  (°C) |
| --- | --- | --- | --- | --- |
| TaMAPK3 | ATGAGCGAGTCTGACCTGGAGTTC | AGTAGTGAGCCTGCCGGGTTCTTC | MAPK | 60℃ |
| TaMAPK12;1 | TCCGGACTTCTGCTCAGGGTATTA | CGGCGCGACATTTTTCAACT | MAPK | 60℃ |
| TaMAPK16 | GCCGCGAGTTCCGTGACATCTACA | GAGCAAGGCCGAAGTCGCAAATCT | MAPK | 60℃ |
| TaSIM | AACGCCGTCAAGAATCACT | GAAGAAACCGCCACCACTA | TF | 60℃ |
| TaNAC2 | ATCGGCAGCGGAGCGATT | AGGGGTCGAAGCGGTAGAGG | TF | 60℃ |
| TabZIP15 | CAAATCATCAGATACCAGGCGA | CCTGTGCCTGTGAGAACGCT | TF | 60℃ |
| TaPP2C-a7 | AGTCCCACTTCTTCGGCGTCT | CTTCTCGTGCTCCTCCGCCACC | PP2C | 60℃ |
| TaPP2C-a10 | CCGCTCTCCCAGGACCACAAGC | CGTCGTCCTCGCACCTTTCGGTT | PP2C | 60℃ |
| TaPP2C-30 | CACTCTCATCCGACCACAAG | AATGGCTTCAGGTAGCTGTC | PP2C | 60℃ |
| TaSnRK2.9 | TGGATGACCTGGACTCGG | AAGCAAACAAGGTGGAAGAC | SnRK2 | 60℃ |
| TaABA 8'-OH1 | ACAGATGGTCCACCTCCAAG | CCTCTATCGTGCCGTTGATT | ABA 8'-OH | 60℃ |
| TaGAPDH | TTCAACATCATTCCAAGCAGCA | CGTAACCCAAAATGCCCTTG | Housekeeping gene | |

Table S4 Taxonomy of drought-responsive OTUs in bulk soil and rhizosphere.

Note: Table S4 is in another Supplemental Material.


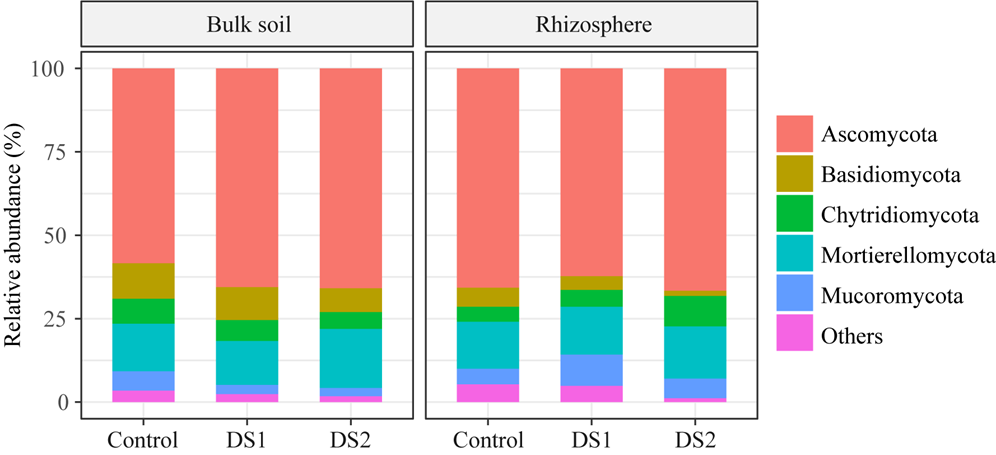


Fig. S1 Composition of fungal communities at phylum level.


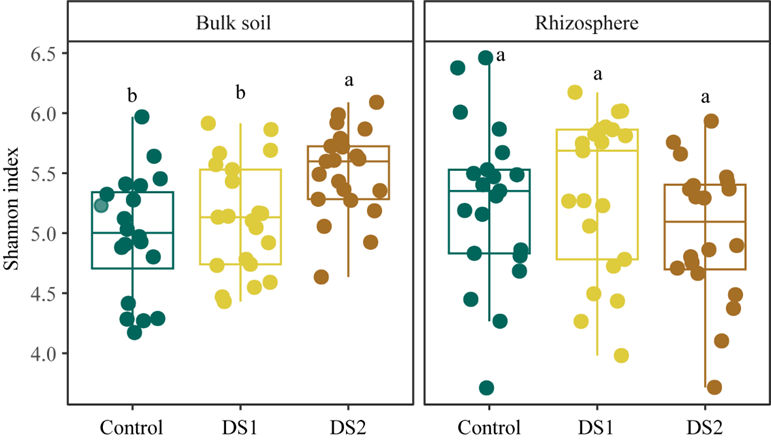


Fig. S2 Alpha diversity of fungal communities (Shannon index) among different treatments in bulk soil and rhizosphere. Statistical analysis is performed among control, DS1 and DS2 by one-way ANOVA and Tukey’s HSD test.


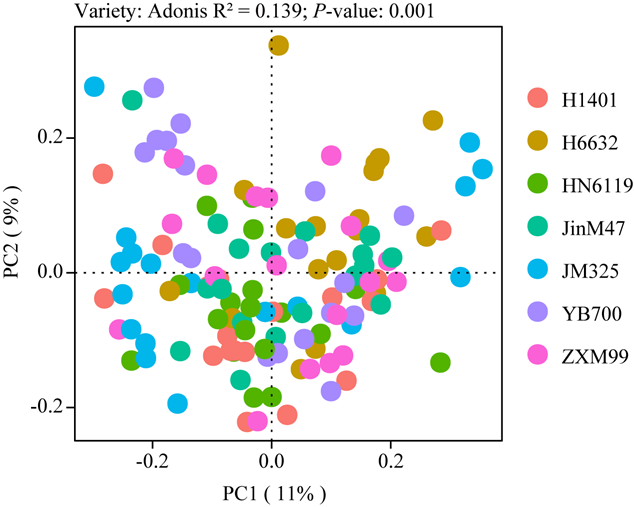


Fig. S3 Unconstrained PCoA with Bray-Curtis distance showing that the fungal communities separate among wheat varieties (*P* < 0.001, permutational multivariate analysis of variance (PERMANOVA) by Adonis).


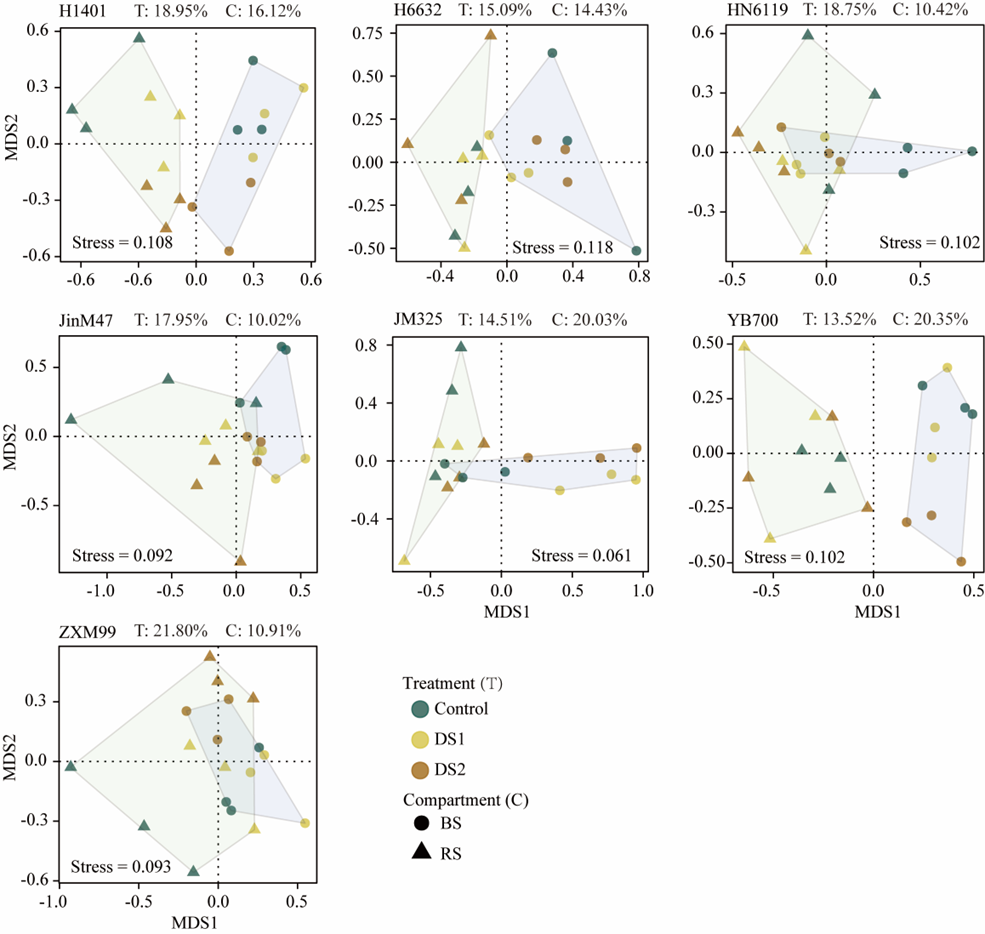


Fig. S4 Drought treatment and soil compartment significantly shape the overall composition of fungal communities in each variety (*P* < 0.001, permutational multivariate analysis of variance (PERMANOVA) by Adonis).


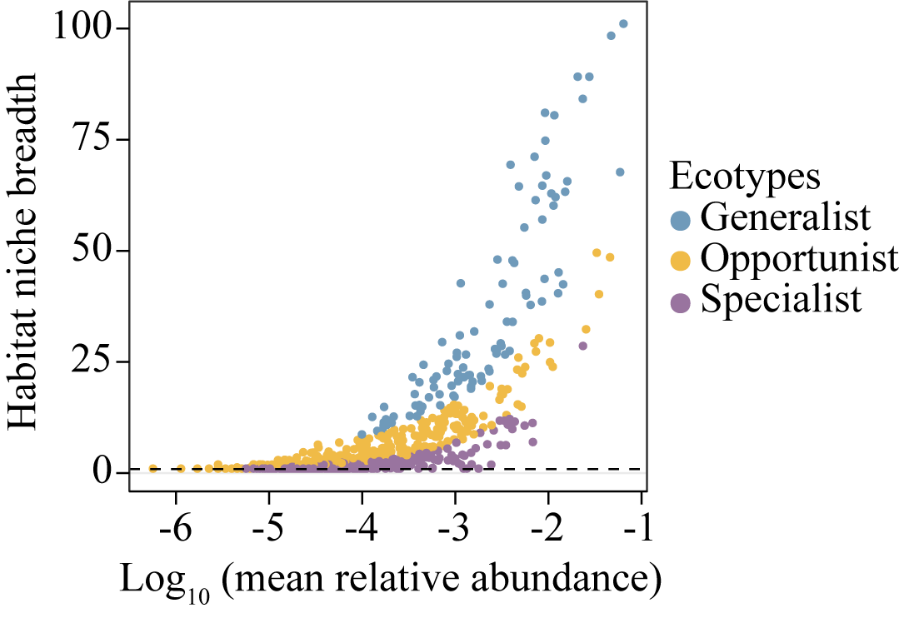


Fig. S5 Habitat niche breadth of the specialists, opportunists, and generalists. The X-axis indicates average relative log-abundances. Each dot represents an individual OTU.


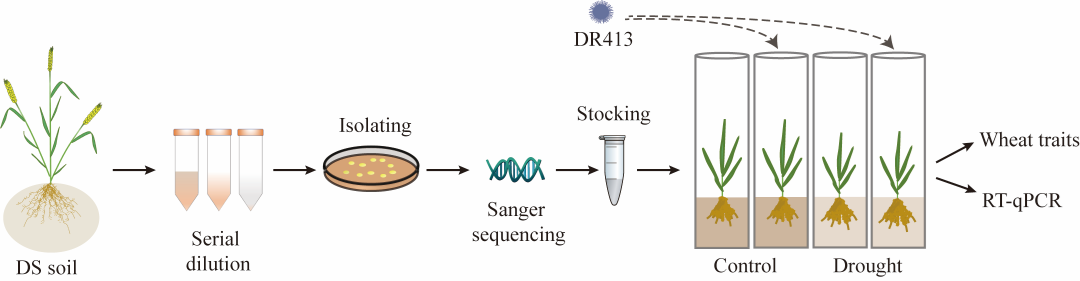


Fig. S6 Schematic representation of the procedures for isolating the fungal strain, and processes to evaluate the impact of *Chaetomium* sp. DR413 (DR413) on wheat growth.


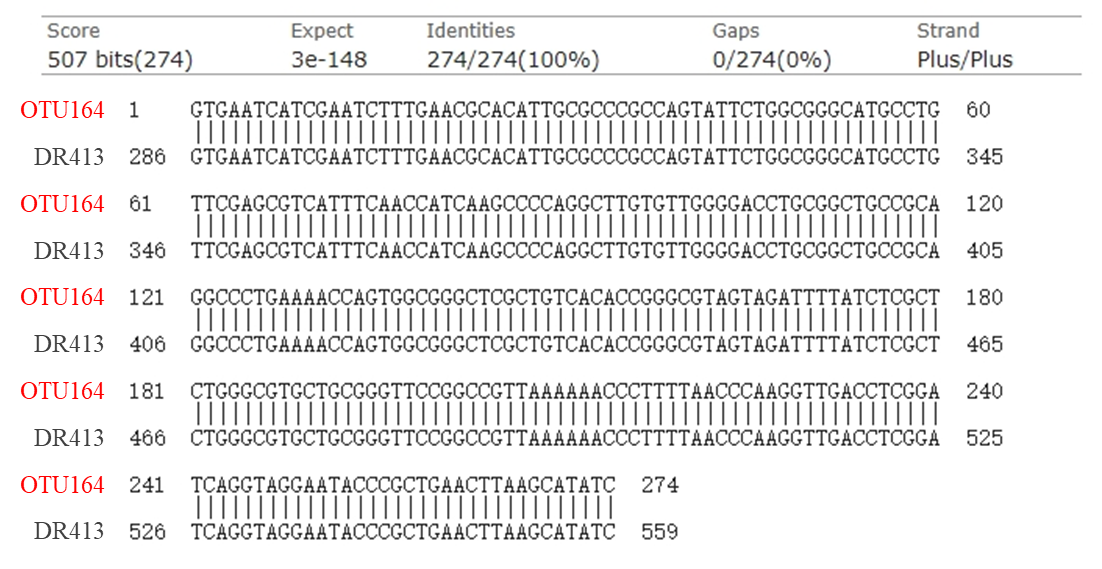


Fig. S7 Nucleotide similarity comparison between the sequences of OTU164 and *Chaetomium* sp. DR413 based on NCBI BLAST.


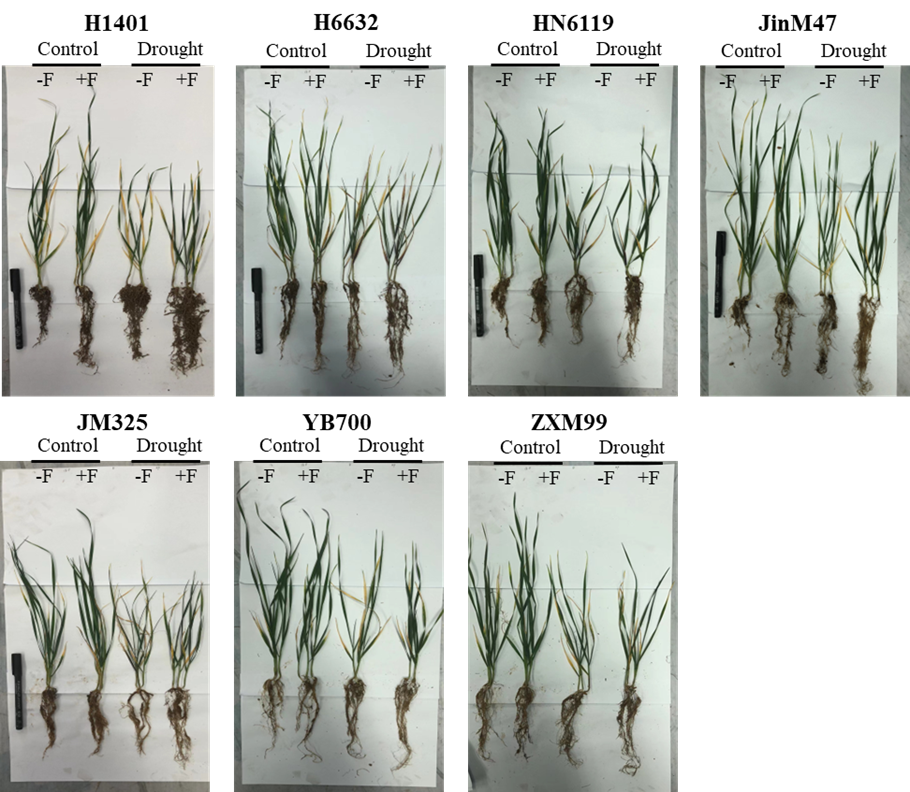


Fig. S8 Picture showing the harvested wheat plants grown in control and drought-stressed soils with (+F) or without (-F) inoculation of *Chaetomium* sp. DR413.


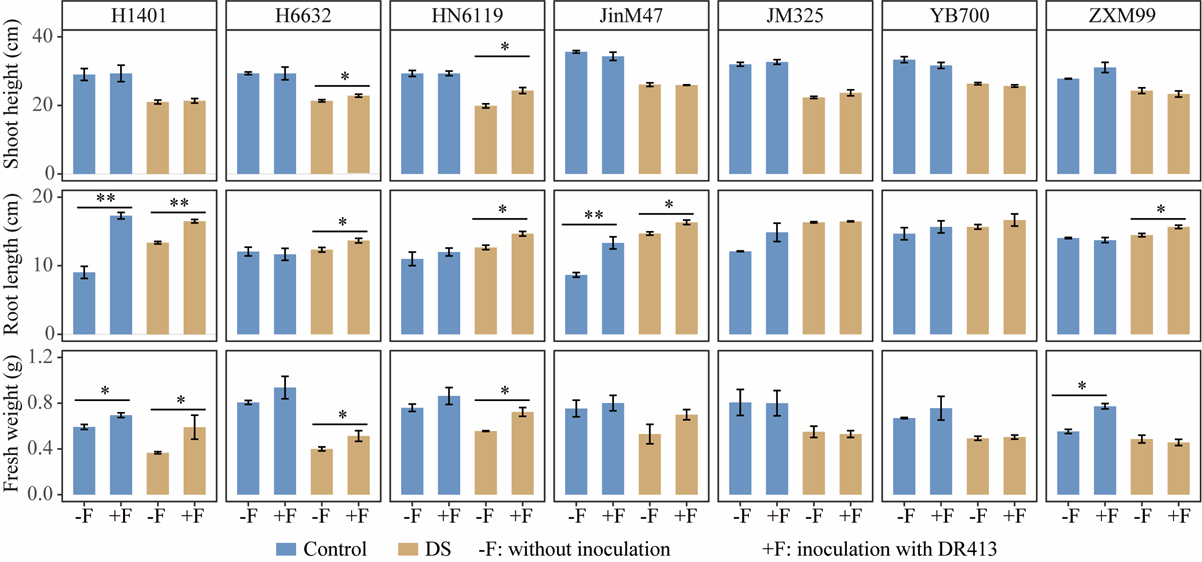


Fig. S9 Effects of Chaetomium sp. DR413 inoculation on plant shoot height, root length and fresh weight in control and drought (DS) treatments. The data of each bar is expressed as the mean ± SD (n = 3), and the asterisk indicate significance (**P* < 0.05, ***P* < 0.01).


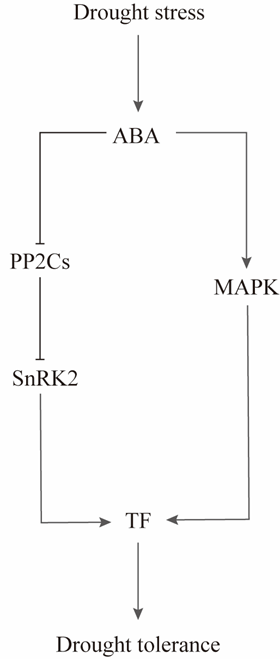


Fig. S10 Schematic representation of ABA signaling pathway during drought stress. Under stresses, the cellular concentration of ABA increases, leading to the binding of PYR/PYL/RCARs and subsequent deactivation of PP2Cs. The SnRK2s is activated when they dissociate from PP2Cs, and phosphorylate downstream targets and trigger TFs. The MAPK cascade also occurs in response to increased ABA levels.


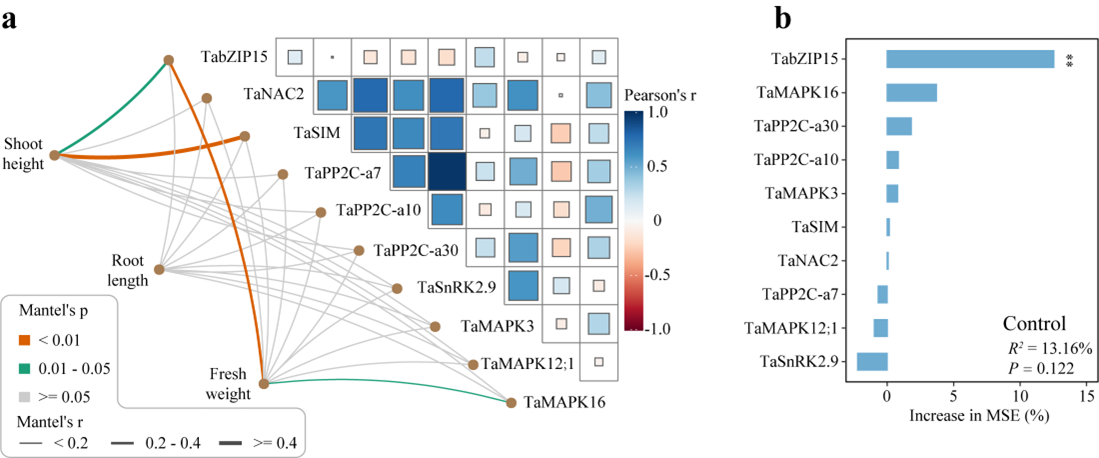


Fig. S11 a, Mantel’s correlations between wheat shoot height, root length and fresh weight with ABA signaling pathway genes in control treatment. Edge width corresponds to the Mantel’s r statistic, and edge color denotes the statistical significance. c, Contribution of ABA signaling pathway genes in predicting wheat root length in control treatment based on random forest modelling analyses. **P* < 0.05, ***P* < 0.01. MSE, mean square error.
